# How I do it: purely intracondylar approach to the hypoglossal canal for the treatment of an intracanalicular hypoglossal lesion

**DOI:** 10.1007/s00701-025-06629-x

**Published:** 2025-09-20

**Authors:** Amedeo Piazza, Simone Olei, Maria Peris-Celda, Carlo Serra

**Affiliations:** 1 Division of Neurosurgery, Sapienza University of Rome, Rome, Italy; 2 Department of Neurologic Surgery, Mayo Clinic, Rochester, USA; 3 Mayo Clinic Rhoton Neurosurgery and Otolaryngology Surgical Anatomy Program, Rochester, MN, USA; 4 Department of Neurosurgery, University Hospital Zurich, 8091 Zurich, Switzerland; 5 Department of Biomedical Sciences, Humanitas University, Via Rita Levi Montalcini 4, 20072 Pieve Emanuele - Milan, Italy; 6 Clinical Neuroscience Center, University Hospital Zurich, University of Zurich, 8057 Zurich, Switzerland

**Keywords:** Skull base, Hypoglossal nerve, MIS surgery, Keyhole surgery, Posterior fossa

## Abstract

**Background and importance:**

Lesions confined to the hypoglossal canal are rare, but may lead to invalidating symptoms. Moreover, extensive approaches to this region frequently require complex dissections and high surgical risks. We propose a minimally invasive approach specifically tailored to purely intracanalicular lesions of the hypoglossal canal.

**Clinical presentation:**

With the aid of operative images and anatomic dissection, we present a clinical case of such a lesion and describe a targeted surgical approach for treatment.

**Conclusion:**

For lesions purely confined to the hypoglossal canal, a tailored intracondylar approach is feasible and may, in selected cases, reduce surgical times and complications.

**Supplementary Information:**

The online version contains supplementary material available at 10.1007/s00701-025-06629-x.

## Relevant surgical anatomy

Lesions purely confined to the hypoglossal canal are rare and mostly consist of intracanalicular schwannomas, frequently causing hypoglossal nerve palsy [2]. Management of such lesions frequently requires extensive approaches, including the far-lateral transcondylar [7], the far-medial endoscopic endonasal [5] or the fully endoscopic transtubercular [1, 3, 4]. These approaches require extensive neck dissections or complex dissections involving the nasal and epipharyngeal regions. This may prolong surgical time and increase the risk of complications. For such small lesions limited to the hypoglossal canal, we propose a less invasive approach and preliminary demonstrate its feasibility by anatomic dissections.

Following approval by our Institutional Review Board (Protocol number 17–005898), dissections were performed in 2 embalmed human cadaver heads (4 sides). The specimens were embalmed and arteries and veins were injected with latex using a 6-vessel technique. The specimens were dissected using operative microscope (© 2024 Leica Microsystems), 4-mm 0- and 30-degree rigid endoscopic lenses (Stryker Corporation, Kalamazoo, Michigan, United States), a high-speed surgical drill with endoscopic compatibility (Medtronic Midas Rex electric system; Medtronic USA Inc., Jacksonville, Florida, United States) and standard endoscopic instrumentation.

**Fig. 1:**
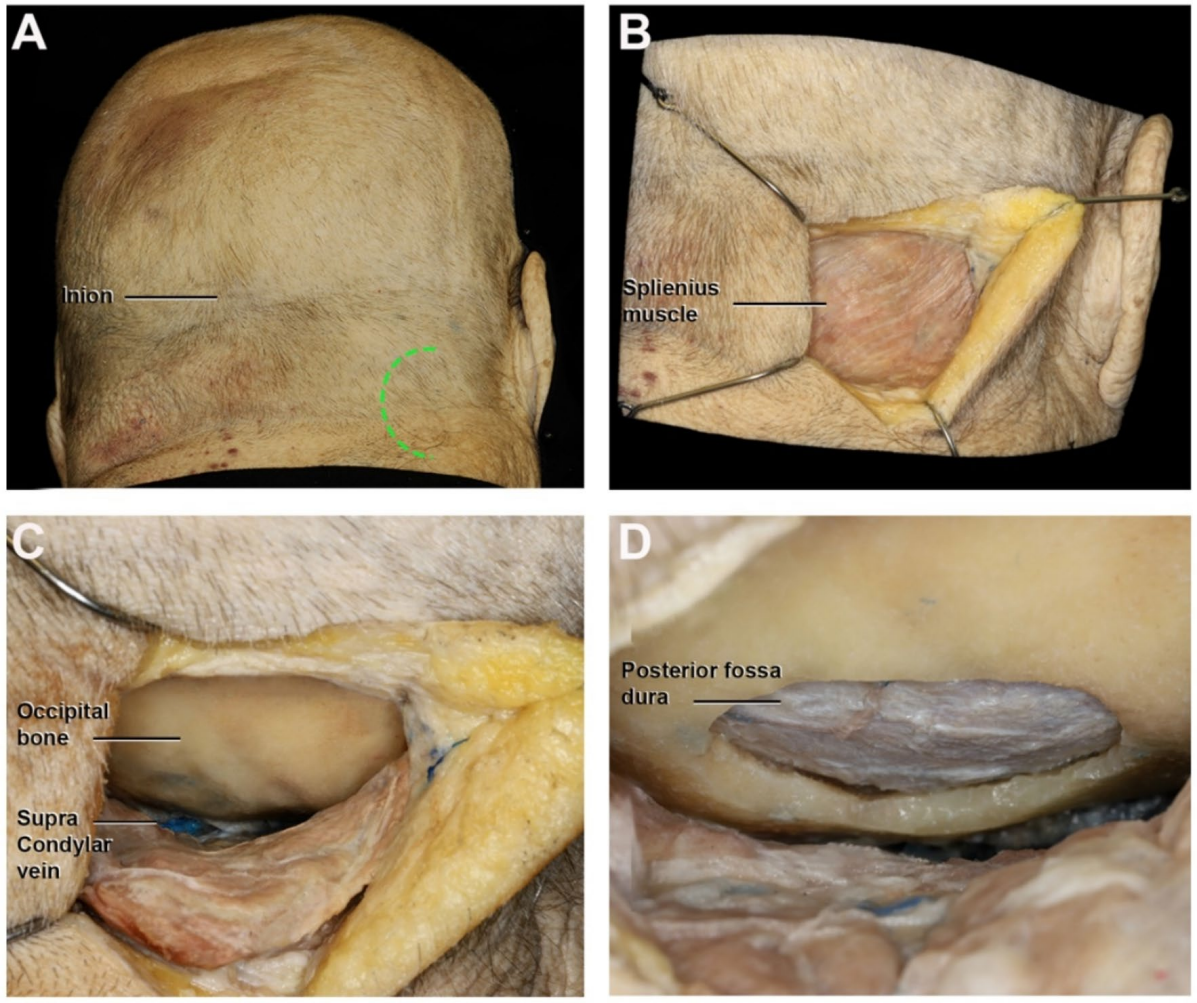
Photographs of cadaveric dissection showing the step-by-step approach to the hypoglossal canal: **A**. The green dotted line indicates the skin incision. **B**. The subcutaneous dissection reveals the splenius muscle. **C**. Subsequent one-layer muscle dissection reveals the occipital bone. The landmark for the occipital condyle is represented by the supracondylar vein. **D**. The craniectomy with a diameter of 3 cm is shown

**Fig. 2:**
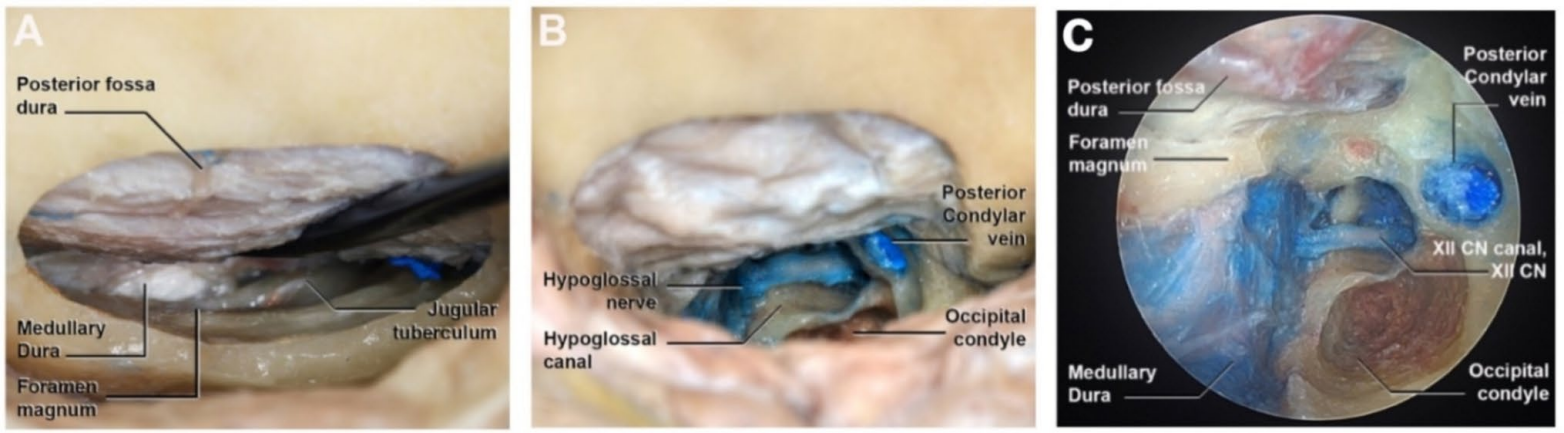
Photographs of cadaveric dissection. **A**. After subperiosteal dissection, the jugular tuberculum is shown laterally to the foramen magnum and the medullary dura. **B**. Direct view of the XIIth cranial nerve after unroofing the hypoglossal canal and partial drilling of the occipital bone. The occipital condyle marks the posterior-inferior limit of the hypoglossal canal. **C**. Endoscopic view of the final stage of the approach in the anatomic specimen, clarifying the relationship between condyle and hypoglossal canal

A 5 cm vertical C-shaped incision was performed, with medial convexity reaching 2 cm from the midline. The inferior limit of the incision laid at the level of the tragus, while the superior margin reached halfway along the length of the auricular pinna (Fig. 1A). Subsequent dissection of the subcutaneous tissues revealed the splenius capitis muscle and the posterior band of the sternocleidomastoid muscle (Fig. 1B). A single-layer subperiosteal muscle dissection exposed the occipital bone. Further dissection of the craniovertebral junction unveiled the supracondylar vein, serving as a landmark for the occipital condyle, situated inferolateral to the vein (Fig. 1C). A paramedian lateral suboccipital craniotomy with a diameter of 3 cm was performed (Fig. 1D). Lateral to medial dissection of the dural layer allowed visualization of the posterior-lateral border of the foramen magnum, the jugular tuberculum and the medullary dura (Fig. 2A). The hypoglossal canal was exposed and unroofed by drilling the occipital bone and partially removing its posteromedial border. This created a surgical corridor with superior-posterior unroofing of the hypoglossal canal. As depicted in Figs. 2B and C, the entire hypoglossal canal can be successfully exposed.

**Fig. 3:**
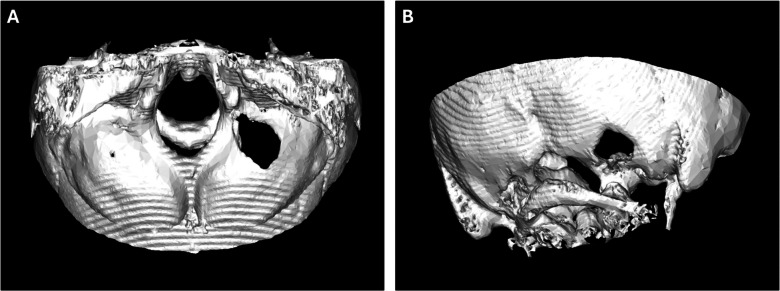
Postoperative 3D reconstruction. Axial (**A**) and oblique (**B**) views illustrate the extent of bone removal and minimal condylar drilling, preserving craniovertebral junction stability

This approach provides adequate exposure of the hypoglossal nerve while minimizing condylar drilling, thereby reducing the risk of craniovertebral junction instability (Fig. 3).

## Description of the technique

### Clinical case

**Fig. 4:**
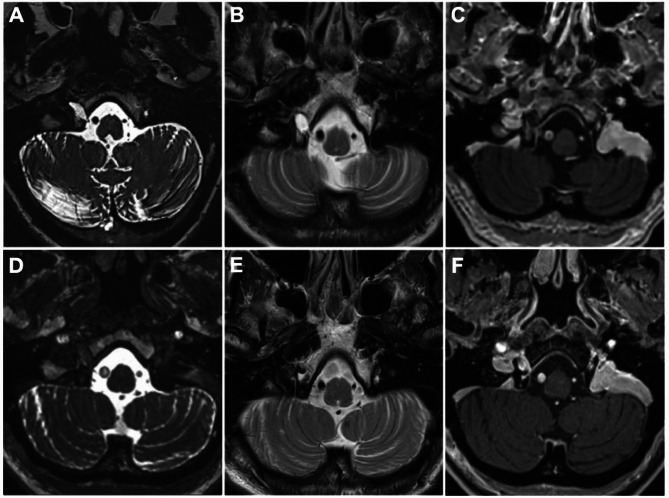
Pre- and postoperative MRI images. **A**. Preoperative CISS sequence. **B**. Preoperative T2 sequence. **C**. Preoperative contrast-enhanced T1 sequence. **D**. Postoperative CISS sequence. **E**. Postoperative T2 sequence. **F**. Postoperative contrast-enhanced T1 sequence. After surgery, the intracanalicular lesion is substantially disappeared

A 76-year-old woman presented with right-sided hypoglossal nerve palsy and tongue atrophy. The symptoms conditioned a slight dysarthria and difficulty in initiating swallowing. Remaining neurologic examination was unremarkable. At MRI, a clear-margin mass with cystic appearance involving the intracanalicular portion of the hypoglossal nerve with minimal cisternal involvement was identified. The hypoglossal canal appeared enlarged and stuffed by the lesion, showing uniform T1 hypointensity and T2 hyperintensity. After contrast administration it showed a slow, minimal, homogeneous enhancement without nodules (Fig. 4A-C). Surgical indication was given due to the negative impact of the symptoms on the patient’s Quality of Life and progressively increasing of dysphagia and dysarthria. Preoperative contrast-enhanced MRI revealed ipsilateral dominance of the vertebral artery and contralateral dominance of the sigmoid sinus.

### Procedure

**Fig. 5:**
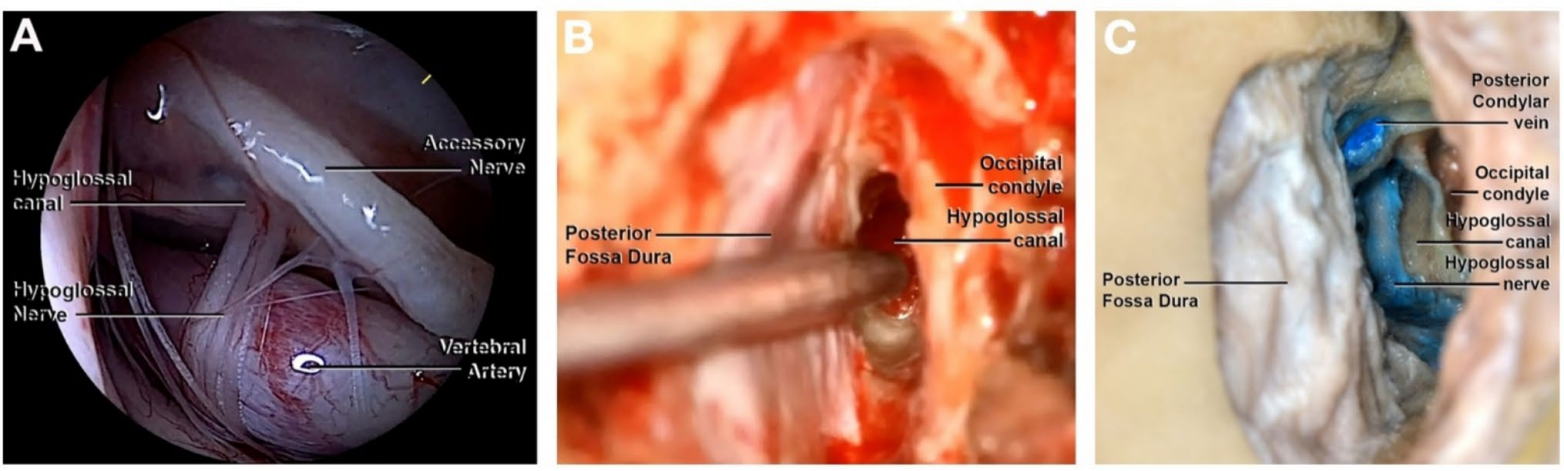
Operative view. **A**. Intradural endoscopic view of the hypoglossal nerve reaching its canal. The vertebral artery, dominant on this side, occupies a great part of the field and is seen in tight contact with the nerve. **B**. Microscopic view of the surgical corridor reaching the lesion in the hypoglossal canal. **C**. Cadaveric dissection paralleled to the intraoperative view, demonstrating the anatomy of the approach

The procedure was performed utilizing neuronavigation and neuromonitoring. After left-sided lateral positioning with head secured to a 3-pin Mayfield holder, the approach described above was performed until craniotomy. A 1 cm dural incision was made caudally to release CSF from the cisterna magna and relax the cerebellum. A further 2 cm long dural incision was performed at the lateral border of the craniectomy to allow insertion of a 30° endoscope (2 mm diameter) and inspection of the entrance to the hypoglossal canal, showing only minimal cisternal extension of the lesion. The dominant vertebral artery was visualized, traveling close to the XIIth nerve (Fig. 5A). The hypoglossal canal was then reached via a purely intracondylar approach restricted within the cortical boundaries of the condyle itself, by drilling the cancellous bone with a high-speed drill under continuous irrigation. Neuronavigation was helpful in directing the working angle, whereas the tactile feedback determined by the cortical bone surrounding the hypoglossal canal proved to be a guiding element in determining the depth of drilling. After approaching the canal (Fig. 5B), its *dura propria* was opened. The cystic lesion was contextually fenestrated. Incision of the cyst released a small amount of clear, slightly yellowish fluid, which allowed for relaxation of the cyst walls and subsequent microsurgical resection. After accurate hemostasis, watertight dural closure was performed. Closure of the muscle and skin layers was performed meticulously. Throughout the procedure, neuromonitoring showed stability of MEPs and SSEPs. At the end of the procedure, direct stimulation demonstrated integrity of the hypoglossal nerve. The total operative duration time was 207 min.

A postoperative MRI demonstrated complete removal of the lesion and hypoglossal nerve decompression without complications (Fig. 4D-F).

Postoperative clinical course proved unremarkable. According to histopathological analysis, the excised tissue was compatible both with an intracanalicular hypoglossal schwannoma and an intracanalicular arachnoid cyst, but no univocal judgement was possible. Classic histological schwannoma stainings, such as SOX-10 and S100, were absent.

At the six-month follow-up after logopedic rehabilitation, the patient showed a significant reduction in dysphagic symptoms and partial improvement in dysarthria.

## Indications

This minimally invasive approach is specifically tailored to purely intracanalicular lesions of the hypoglossal canal, possibly simplifying surgery and reducing the risk of complications in cases presenting without extensive cisternal involvement.

## Limitations

This tailored surgical approach effectively exposes the area of the hypoglossal canal, reducing surgical time and minimizing operative aggressiveness. Nevertheless, we believe that extensive approaches should be contemplated for intracanalicular masses in patients with ipsilateral tortuous vertebral artery and/or ipsilateral dominant jugular bulb, owing to a significant reduction of surgical space and potential difficulty in managing complications. Therefore, preoperative angio-MR, together with routine preoperative CT and MRI imaging, should be considered in assessing the feasibility of this approach.

The far-lateral approach and its variants afford extensive exposure of lesions situated posterolateral and anterolaterally to the craniovertebral junction, while the supracondylar approach exposes the region located posteriorly to the jugular foramen. Conversely, the far-medial endoscopic endonasal approach targets lesions anterior and anterolateral to the craniovertebral junction, and anterior to the jugular foramen [1]. Both these approaches are preferable for large intradural and extradural masses [6]. However, due to the limited exposure area confined to the hypoglossal canal, this approach appears particularly suitable for small, purely intracanalicular lesions. Caution is advised to avoid overtreatment.

## How to avoid complications

A tailored approach is suitable for purely intracanalicular lesions with no or only minimal cisternal involvement. For larger lesions, the risk of damaging the sigmoid sinus and the vertebral artery in a strict corridor should lead to choosing a wider approach.

The presence of a dominant sigmoid sinus ipsilateral to the lesion entails a greater risk of sinus damage. A tortuous ipsilateral vertebral artery (V4 segment) may hinder surgical maneuverability and entails a greater risk of arterial damage. In this case, a wider approach may be preferable.

The use of the neuronavigation system can guide the approach and reduce operative risks.

## Specific information for the patient

Despite the lower invasiveness of the approach, the risks connected with sigmoid sinus and vertebral artery damage must be taken into consideration. Injury to either structure carries the potential for severe complications, such as brainstem ischemia or ascending venous thrombosis.

Postoperative hematoma, CSF leakage and infection are possible.

## Ten Key Points Summary

- Purely intracanalicular hypoglossal lesions are rare but may cause disabling symptoms.
- Usual treatment involves extensive approaches with potentially high surgical risks, such as the far-lateral trans-condylar approach, far-medial endoscopic endonasal approach, and fully endoscopic transtubercular approach.
- For Purely intracanalicular with minimal or no cisternal involvement, a minimally invasive intracondylar approach can be considered.
- Preoperative angio-MRI can assess the surgical corridor and associated risks by identifying an ipsilateral dominant sigmoid sinus or a tortuous vertebral artery.
- Anatomical dissections on embalmed cadaver heads confirmed the feasibility of this approach.
- Through a direct intracondylar route, the hypoglossal canal can be accessed and opened without intradural structure manipulation.
- We present the case of a 76-year-old woman with a symptomatic intracanalicular hypoglossal lesion. Indication to surgical treatment was given through a minimally invasive approach.
- An unremarkable postoperative clinical course was achieved along with complete resection.
- The use of the minimally invasive approach significantly shortened the surgical time and minimized the need for dural opening.
- An extensive approach should be considered for every lesion with significant intradural extension.

## Supplementary Information

Below is the link to the electronic supplementary material.

Supplementary file1 (MP4 236333 KB)

## Data Availability

No datasets were generated or analysed during the current study.
